# Comparison of short-term clinical efficacy between shoulder arthroplasty and plate fixation combined with bone cement augmentation for pathological fractures of the proximal humerus caused by metastatic tumors: a retrospective study of 40 cases

**DOI:** 10.3389/fmed.2026.1788879

**Published:** 2026-04-10

**Authors:** Jinxian Zhao, Haiwen Pan, Jianfeng Chen, Guokai Feng, Zongquan Mo, Yongqiang Lao

**Affiliations:** 1 The Eighth Clinical Medical College, Guangzhou University of Chinese Medicine, Guangzhou, China; 2 Foshan Hospital of Traditional Chinese Medicine, Foshan, China

**Keywords:** bone cement, locking plate, pathological fracture, proximal humerus, shoulder arthroplasty

## Abstract

**Background:**

The proximal humerus is an easily overlooked site for metastatic bone tumors, and pathological fractures here can cause severe pain and functional impairment. The selection of surgical regimens should take into account the location of bone metastases, the patient’s physical status, and other relevant factors. Therefore, the optimal surgical strategy remains controversial. This study aimed to investigate and compare the clinical efficacy of two surgical approaches—tumor resection combined with prosthesis implantation versus plate fixation combined with bone cement augmentation—in patients with pathological fractures of the proximal humerus induced by metastatic tumors. By evaluating functional recovery, local tumor control, complications, and survival rate, this study clarified the applicable scenarios of the two surgical modalities, thereby providing a reference for clinical treatment decision-making.

**Methods:**

A retrospective analysis was performed on 40 patients with metastatic pathological fractures of the proximal humerus who were admitted to our institution from July 2017 to May 2025 (24 males and 16 females, with a mean age of 57 years). According to the surgical procedures, the patients were divided into two groups: the prosthesis group (*n* = 15) underwent tumor resection combined with modular anatomical hemiarthroplasty, while the plate-cement group (*n* = 25) was treated with open reduction, plate internal fixation plus bone cement augmentation. Baseline data and perioperative indicators including operation time, intraoperative blood loss, and incision length were collected and compared between the two groups. In addition, postoperative pain assessed by the Visual Analogue Scale (VAS), functional outcomes evaluated via the Musculoskeletal Tumor Society (MSTS) score, postoperative complications, and survival rate were also compared between the two cohorts. The mean follow-up duration was 13.03 ± 19.59 months.

**Results:**

A total of 40 patients were enrolled in this study, consisting of 15 cases in the prosthesis group and 25 cases in the plate-cement group. No significant differences were observed in gender, primary tumor type and fracture type between the two groups (*p* > 0.05). With respect to perioperative indicators, the plate-cement group showed significantly superior outcomes to the prosthesis group in terms of intraoperative blood loss (374.00 ± 100.13 mL vs. 973.33 ± 749.49 mL, *p* < 0.05) and operation duration (93.40 ± 20.19 min vs. 146.87 ± 17.65 min, *p* < 0.05). At the 3-month postoperative follow-up, the plate-cement group achieved significantly lower VAS pain scores (0.72 ± 0.80 vs. 1.27 ± 0.80, *p* < 0.001) and significantly higher MSTS functional scores (27.20 ± 1.38 vs. 21.53 ± 1.19, *p* < 0.001) compared with the prosthesis group. Regarding complications during the short-term follow-up, two cases of shoulder instability occurred in the prosthesis group, whereas the plate-cement group presented with one case of humeral head collapse and two cases of transient radial nerve injury.

**Conclusion:**

Plate fixation with bone cement augmentation provides superior short-term functional recovery and pain relief for metastatic proximal humeral fractures. However, these findings must be interpreted with caution due to the selection bias inherent in our non-randomized surgical approach.

## 1 Introduction

Metastatic bone tumors are the most common malignant bone tumors in adults, and the humerus is the second most frequent long bone site for metastasis after the femur, with an incidence of 16 to 27% reported in the literature (1). Pathological fractures or impending pathological fractures of the proximal humerus can lead to severe pain, significant loss of function, and severely impaired quality of life and self-care ability in patients. Conservative treatment often results in progressive tumor invasion and poor prognosis. The goals of surgical treatment include controlling tumor extent, relieving pain, and restoring limb stability to enable early upper limb use and improve quality of life. Current main surgical options include intramedullary nailing, plate fixation combined with bone cement augmentation, and shoulder arthroplasty (2). When the tumor invades the humeral head articular surface or causes extensive bone destruction of the proximal humerus, shoulder arthroplasty is considered the standard procedure for joint function reconstruction. Its advantages include complete tumor resection and immediate rotational and flexion stability. However, this procedure is associated with large surgical trauma, prolonged postoperative immobilization, and potential risks such as shoulder dysfunction and prosthetic loosening (3). For patients with tumors confined to the humeral neck or proximal metaphysis with an intact articular surface, locking plate fixation combined with bone cement augmentation provides a joint-preserving option. This technique achieves stable fixation of the neck-shaft angle, fills bone defects with bone cement to achieve both immediate stability and tumor devitalization, and allows early postoperative functional exercise (4). Although each technique has its indications, the choice of surgical approach for lesions involving the humeral head–neck junction is often controversial and overlapping. The core of the controversy lies in balancing the three therapeutic goals: “local tumor control”, “long-term mechanical stability,” and “shoulder joint function preservation.” Plate fixation tends to provide short-to-medium-term stability through intralesional curettage, bone cement filling, and internal fixation while maintaining the anatomical structure and function of the shoulder joint (5). Its advantages include relatively simple surgery and preservation of native joint function, but it carries risks of local recurrence and long-term internal fixation failure (6). Prosthetic replacement achieves more thorough local control and permanent mechanical stability through extensive tumor segment resection (7, 8), but it sacrifices the complex active motion function of the shoulder joint and is associated with large surgical trauma and long-term prosthetic-related complications. The underlying reasons for this controversy stem from the uncertainty of multiple variables, such as tumor biological behavior (e.g., sensitivity to radiotherapy and chemotherapy), the mechanical extent of bone defects, expected survival time, and individual functional needs. Therefore, there is no unified standard for clinical decision-making, and comprehensive consideration of individual factors (e.g., patient age, comorbidities, and lesion extent) is required (9). Prosthetic replacement is preferred for patients with a long expected survival time and large tumor burden, while plate fixation is generally recommended for those with a short expected survival time (10). Intramedullary nailing is first recommended for humeral shaft fractures, and the choice between plate-cement fixation and arthroplasty is considered for proximal humeral fractures. Therefore, this retrospective study compared the clinical efficacy of shoulder arthroplasty and plate fixation combined with bone cement augmentation for pathological fractures of the proximal humerus caused by metastatic tumors, aiming to provide evidence for clinical surgical decision-making through literature review and empirical analysis.

## 2 Materials and methods

### 2.1 Study population

A retrospective analysis was conducted on patients with pathological fractures of the proximal humerus caused by metastatic tumors who underwent surgical treatment at our hospital between July 2017 and May 2025. Inclusion criteria: (1) Pathological fractures or impending pathological fractures of the proximal humerus caused by pathologically confirmed malignant tumors; (2) Treatment with shoulder arthroplasty or locking plate fixation combined with bone cement augmentation; (3) Expected survival time ≥3 months; (4) Complete clinical and follow-up data. Exclusion criteria: (1) American Society of Anesthesiologists (ASA) classification Grade 4; (2) Previous ipsilateral shoulder surgery history; (3) Extensive systemic metastasis and poor general condition unable to tolerate surgery. The choice of surgical technique and implant was not randomized but determined by the senior surgeon based on the patient’s estimated survival, tumor burden, and bone stock. Plate fixation with cement augmentation was primarily intended as a palliative stabilization procedure to allow early mobilization, whereas arthroplasty was considered for cases requiring extensive tumor resection.” A total of 40 patients were finally included and divided into the Shoulder Arthroplasty Group (*n* = 15) and the Plate-Cement Group (*n* = 25) based on the surgical method.

This study was approved by the Ethics Committee of Foshan Hospital of Traditional Chinese Medicine (Approval No. KY [2025] R9). Due to the retrospective nature of the study, the requirement for informed consent was waived by the Ethics Committee.

### 2.2 Surgical methods

#### 2.2.1 Shoulder arthroplasty group

**Figure 1:**
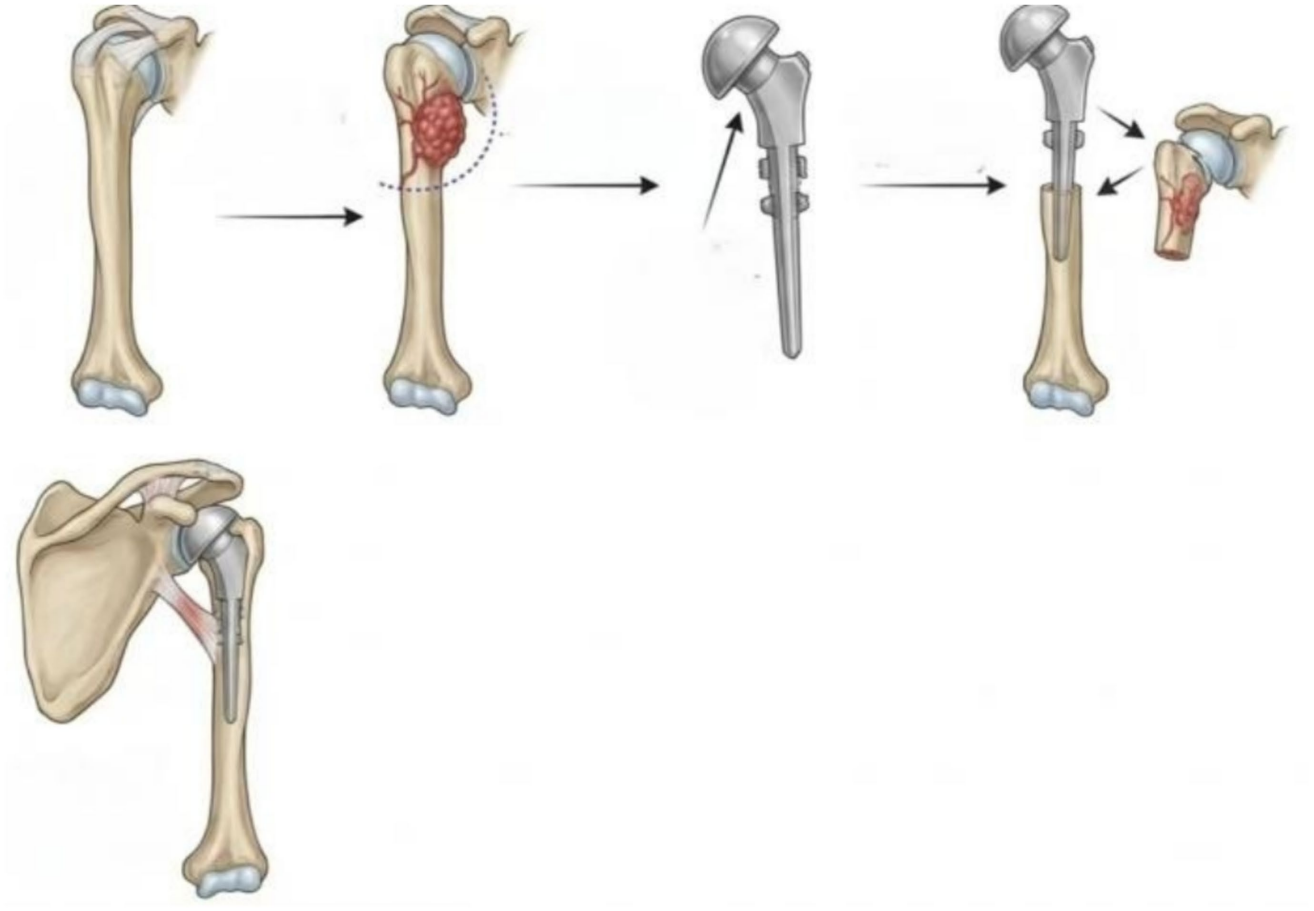
Surgical flowchart of proximal humeral tumor resection and prosthetic reconstruction (1. Normal anatomical structure of the humerus; 2. Extensive local resection of the proximal humeral metastatic tumor; 3. Tumor prosthesis for the right shoulder joint; 4. Prosthesis assembly; 5. Completion of shoulder joint reconstruction).

After successful anesthesia, the patient was placed in the supine position, and routine disinfection and draping were performed. The incision started at the coracoid process, extended proximally via the deltopectoral approach, and distally along the medial edge of the biceps brachii to the lower 1/3 of the upper arm, forming a longitudinal arc incision approximately 15 cm in length. The skin, subcutaneous tissue, and deep fascia were incised layer by layer, the cephalic vein was dissected and retracted laterally for protection, and access was gained through the deltopectoral interval. The proximal humeral tumor was exposed, originating from below the humeral head to above the insertion of the deltoid muscle, with expansile growth. The long head of the biceps brachii tendon was encased by the tumor and was transected outside the tumor and marked. Medially, the pectoralis major, latissimus dorsi, and teres major muscles were transected at their insertions and marked. The axillary sheath was retracted medially for protection. The insertion of the subscapularis muscle was transected at the lesser tubercle superomedially. The rotator cuff was transected superolaterally and marked. The joint capsule was incised circumferentially. Osteotomy was performed approximately 3 cm below the tumor, and the posterior tissues, teres minor muscle, and joint capsule were dissected. The tumor was completely resected en bloc. In this study, all patients in the prosthesis group underwent modular anatomical hemiarthroplasty. No reverse total shoulder arthroplasty (RTSA) or total shoulder arthroplasty (TSA) was performed. Adequate hemostasis was achieved intraoperatively, followed by pulse lavage with a large amount of normal saline. After osteotomy, the length of the tumor segment and the diameter of the humeral head were measured. Next, the distal humerus was reamed, and a prosthetic trial was placed. Once the prosthesis was properly sized, bone cement was injected into the medullary cavity, and the prosthesis was inserted. The elbow was maintained at 90° in the neutral position, with the prosthesis internally rotated by approximately 30°, ensuring that the bicipital groove, midpoint of the distal humerus, and midpoint of the wrist joint were in the same plane. The prosthesis position and soft tissue tension balance were checked. A hernia repair patch was sutured to wrap the proximal humerus. The joint capsule, rotator cuff, pectoralis major, latissimus dorsi, teres major, and long head of the biceps brachii tendon were sutured to the patch. The stability of the shoulder joint was confirmed, the surgical site was irrigated, two drainage tubes were placed in the intermuscular space, and the incision was closed layer by layer to the skin (Figure 1).

#### 2.2.2 Plate-cement group

**Figure 2:**
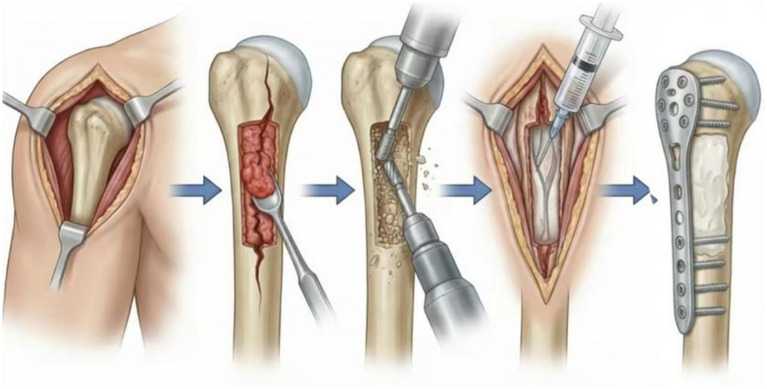
Surgical flowchart of curettage combined with alcohol inactivation and bone cement-plate fixation for humeral pathological tumors (lower panel: 1. surgical exposure of the proximal humerus; 2. tumor curettage through the fracture site; 3. Treatment of the fracture cavity with a high-speed burr; 4. chemical inactivation with 95% alcohol; 5. Bone cement filling combined with plate fixation).

The patient was placed in the supine position. The anterolateral approach to the proximal humerus was used, and the muscular tissue was incised layer by layer. The cephalic vein and coracobrachialis muscle were exposed through the deltopectoral interval, retracted laterally for protection, and the fracture site and tumor area were exposed. The axillary nerve and radial nerve were protected. The tumor lesion was curetted as much as possible with a curette, and the lesion tissue was sent for frozen section examination. The sclerotic area was ground with a high-speed burr, the lesion area was repeatedly electrocoagulated, irrigated thoroughly, and locally devitalized with 95% alcohol for 15 min. Crucially, to prevent tumor seeding, surgical instruments were replaced, and the surgical team performed regloving and re-draping with sterile sheets before reconstruction. Subsequently, antibiotic-loaded bone cement (Palacos® MV + G, Heraeus Medical, 40 g/pack, containing Gentamicin) was filled into the bone cavity. C-arm fluoroscopy confirmed adequate filling of the lesion cavity. An appropriate Zhengtian proximal humeral locking plate was placed 5–8 mm distal to the tip of the greater tuberosity of the humerus and fixed with locking screws, ensuring that at least 6 screws were anchored in the humeral head. After confirming no friction or impingement during shoulder joint movement, a drainage tube was placed, and the incision was closed. The same postoperative protocol was used for both the Plate-Cement Group and the Prosthesis Group. Most patients received adjuvant therapy (Figure 2).

Case 1 Imaging and pathological findings of Case 1 (Male, 56 years old, metastatic squamous cell carcinoma: a–c. Imaging findings of pathological fracture; d. Intraoperative pathological findings; e. Postoperative follow-up findings).


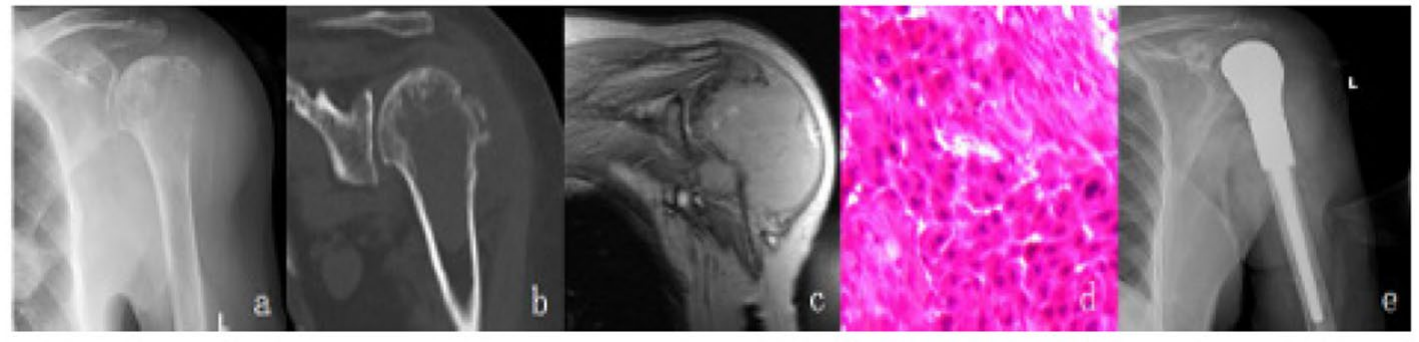


Case 2 Imaging and pathological findings of Case 2 (Male, 51 years old, pathological fracture due to lung cancer metastasis: ①–③. Imaging findings of pathological fracture; ④. Intraoperative pathological findings; ⑤. Postoperative follow-up findings).


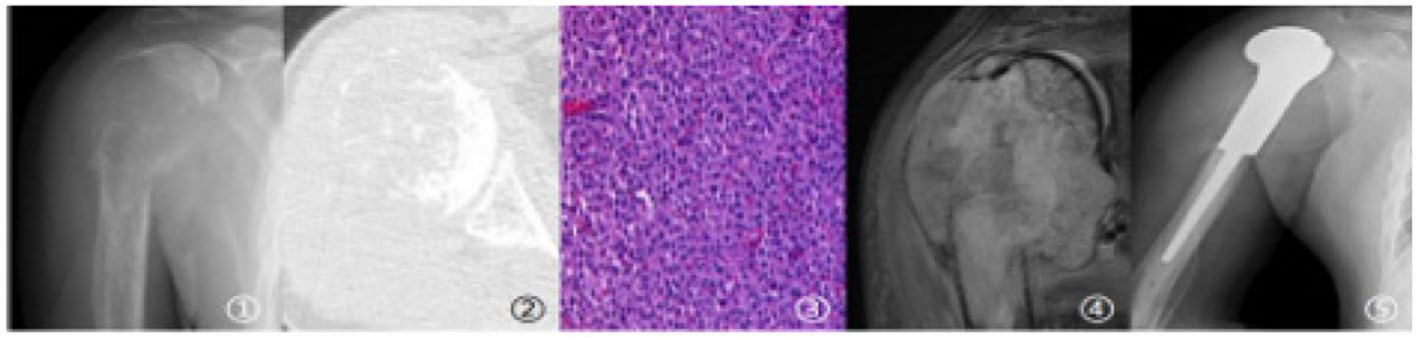


Case 3 Imaging and pathological findings of Case 3 (Male, 54 years old, pathological fracture due to thyroid cancer metastasis: ①–③. Imaging findings of fracture; ④. Intraoperative pathological findings; ⑤. Postoperative follow-up findings).


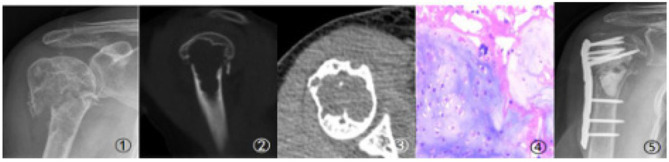


Case 4 Imaging and pathological findings of Case 4 (Female, 59 years old, pathological fracture due to lung cancer metastasis: ①-②. Imaging findings of fracture; ③. Intraoperative pathological findings; ④. Follow-up findings at 3 months postoperatively).


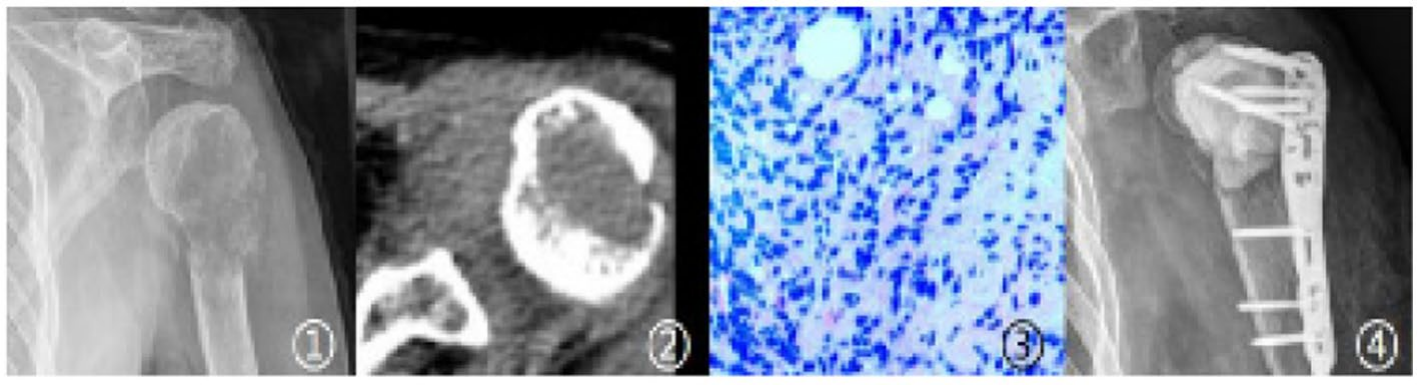


### 2.3 Outcome measures

**Figure 3:**
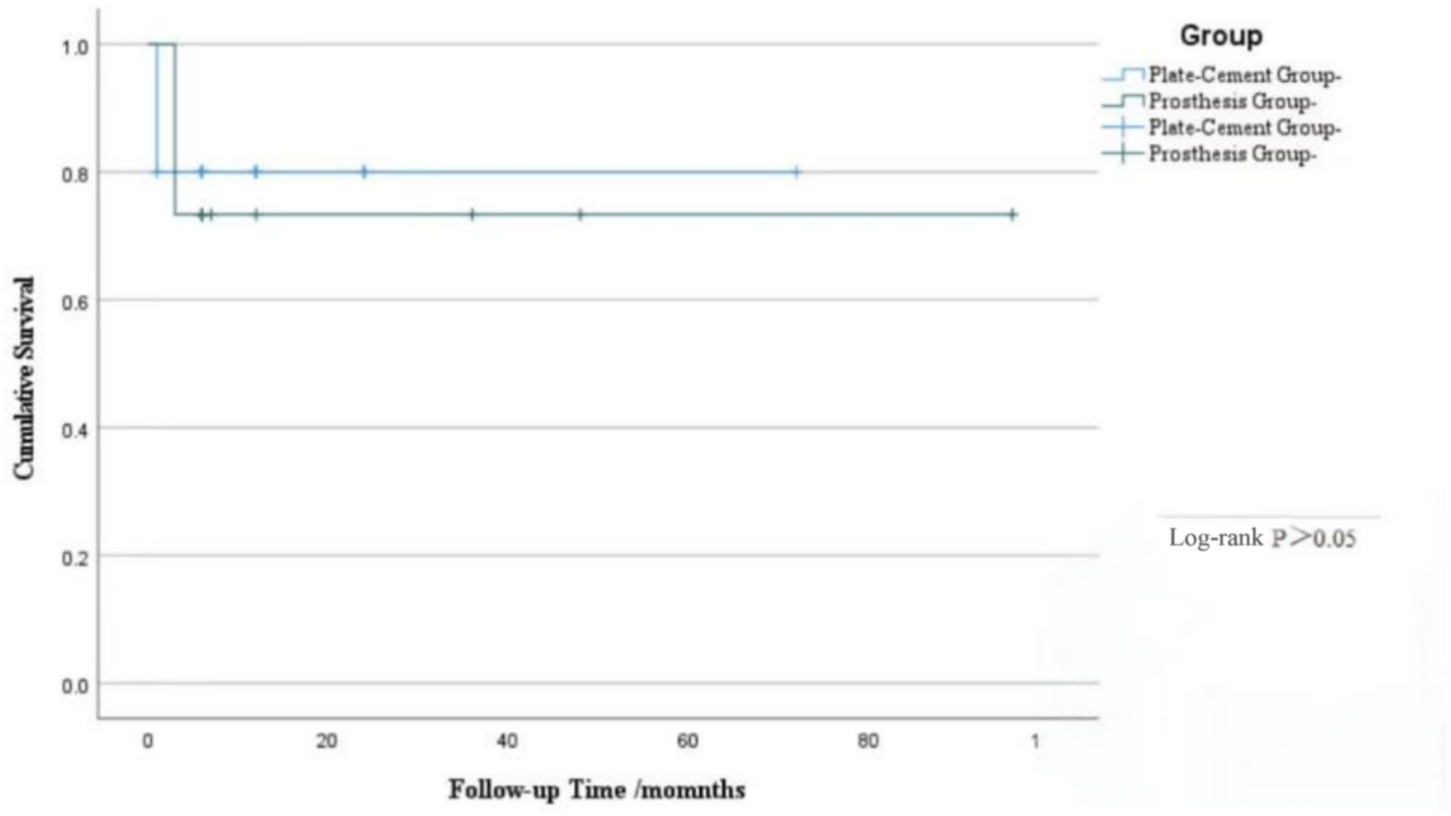
Kaplan–Meier survival curves for overall survival. There was no significant difference in survival rates between the plate-cement group and the prosthesis group (Log-rank *p* > 0.05, Vertical tick marks indicate censored data).

Perioperative indicators: Operation time, intraoperative blood loss, and postoperative hospital stay. Pain assessment: VAS (0–10 points) was used for evaluation preoperatively, postoperatively, and at 3 months. Functional assessment: The MSTS scoring system was used to evaluate upper limb function at 3 months postoperatively. Fracture healing, internal fixation/prosthesis status, and local tumor recurrence were evaluated by X-ray. Complications (infection, nerve injury, shoulder instability “defined as symptomatic subluxation or dislocation,” internal fixation loosening/breakage, prosthetic loosening, bone cement leakage, etc.) were recorded. Patient survival status was confirmed through telephone or outpatient follow-up Figure 3.

### 2.4 Statistical methods

SPSS 27.0 software was used for data analysis. Measurement data were expressed as mean ± standard deviation (SD) or median (interquartile range), and intergroup comparisons were performed using the *t*-test or Mann–Whitney *U* test. Categorical data were expressed as *n* (%), and intergroup comparisons were performed using the *χ*^2^ test or Fisher’s exact test. A *p*-value <0.05 was considered statistically significant.

## 3 Results

### 3.1 Comparison of baseline data

There were no statistically significant differences between the two groups in gender, age, primary tumor type, affected side, or type of pathological fracture (complete/impending) (*p* > 0.05), However, it should be noted that clinical variations in tumor types and lesion locations existed between the groups, reflecting the non-randomized nature of the study (Table 1).

**Table 1:** Comparison of baseline characteristics between the two groups.

Variable	Total (*n* = 40)	Plate-cement group (*n* = 25)	Prosthesis group (*n* = 15)
Age (mean ± SD, years)	57.37 ± 17.78	61.40 ± 13.00	50.67 ± 22.66
Gender, *n* (%)			
Male	24 (60.0)	13 (52.0)	11 (73.3)
Female	16 (40.0)	12 (48.0)	4 (26.7)
Affected side, *n*			
Left	22	12	10
Right	18	13	5
Location of humeral lesion, *n*			
Humeral head	17	5	12
Humeral neck	10	9	1
Subtrochlear to upper humerus	13	11	2
Bone metastasis site, *n*			
Solitary	33	21	12
Multiple	7	4	3
Diagnosis, *n* (%)			
Pathological fracture	40 (100.0)	25 (100.0)	15 (100.0)
Primary tumor, *n*			
Lung cancer	12	10	2
Kidney cancer	2	1	1
Bone marrow tumor	11	1	10
Liver cancer	4	3	1
Others	11	10	1

### 3.2 Comparison of perioperative outcomes

In terms of intraoperative blood loss and operation duration, Group B (the plate-cement group) showed statistically significant superiority over Group A (the prosthesis group), with the differences being statistically significant (*p* < 0.05). Additionally, the postoperative length of hospital stay in Group B was shorter than that in Group A, and the difference was also statistically significant (*p* < 0.05) (Table 2).

**Table 2:** Comparison of perioperative outcomes and functional scores between the two groups.

Variable	Total (*n* = 40)	Plate-cement group (*n* = 25)	Prosthesis group (*n* = 15)	*p*-value
Operation time (mean ± SD, min)	113.45 ± 32.40	93.40 ± 20.19	146.87 ± 17.65	<0.001
Intraoperative blood loss (mean ± SD, mL)	598.75 ± 542.37	374.00 ± 100.13	973.33 ± 749.49	<0.001
Postoperative hospital stay (mean ± SD, days)	14.08 ± 5.98	12.12 ± 5.33	17.33 ± 5.72	<0.001
Complications, *n*		1 case of humeral head collapse and 2 cases of transient radial nerve injury occurred	2 cases of shoulder instability occurred	
Radial nerve injury	2	2	0	
Plate breakage	1	1	0	
Shoulder instability	2	0	2	
VAS score (mean ± SD)				
Preoperative	7.68 ± 1.02	7.64 ± 0.95	7.73 ± 1.16	0.082
Postoperative	4.53 ± 1.04	4.04 ± 0.84	5.33 ± 0.82	<0.001
3-month follow-up	0.93 ± 0.86	0.72 ± 0.84	1.27 ± 0.80	<0.001
MSTS score (mean ± SD, 3-month follow-up)	25.07 ± 3.07	27.20 ± 1.38	21.53 ± 1.19	<0.001
Follow-up duration (mean ± SD, months)	13.03 ± 19.59	11.08 ± 14.78	16.13 ± 25.80	0.062
Survival >6 months, *n* (%)	30 (75.0)	19 (76%)	11 (73%)	>0.05

### 3.3 Comparison of pain and functional outcomes

Three months postoperatively represents a key time point for evaluating early rehabilitation. The plate-cement group exhibited significantly lower VAS pain scores than the prosthesis group (0.72 ± 0.84 vs. 1.27 ± 0.80, *p* < 0.001), indicating superior pain control efficacy. In terms of functional recovery, the plate-cement group achieved markedly higher Musculoskeletal Tumor Society (MSTS) scores compared with the prosthesis group (27.20 ± 1.38 vs. 21.53 ± 1.19, *p* < 0.001). These findings suggest that the joint-preserving surgical approach can preserve better upper extremity function in the short term Table 3.

**Table 3:** Summary of literature on surgical treatments for metastatic humeral fractures.

Author	Time	Sample size	Plate	IMN	Arthroplasty	Complications	Follow-up time
Janssen et al.(11)	2016	134	30	57	N/A	Visceral metastasis: 124 cases (42%); Multiple bone metastases: 222 cases (75%)	Median 11 months
Wedin et al. (12)	2016	69	N/A	29	35	N/A	Median 23 months
Hoellwarth et al. (24)	2020	64	11	39	14	N/A	N/A
Casadei et al. (25)	2018	35	6	N/A	29	N/A	Mean 23 months
Rovere et al. (13)	2022	38	N/A	18	20	N/A	N/A

### 3.4 Comparison of complications

Mean follow-up data of 13.03 ± 19.59 months were obtained in this study, with no statistically significant difference between the plate-cement group (11.08 ± 14.78 months) and the prosthesis group (16.13 ± 25.80 months, *p* = 0.062), confirming comparable follow-up durations between the two cohorts. During the follow-up period, the two groups showed comparable overall survival rates (proportion of patients with survival time >6 months: 76% in the plate-cement group vs. 73% in the prosthesis group). This observation suggests that the selection of surgical modality does not exert a significant impact on patients’ tumor prognosis.

### 3.5 Comparison of complications

In Group A (the prosthesis group), one case developed deep periprosthetic infection, which was controlled after debridement and drainage; two cases presented with shoulder subluxation, and the symptoms were ameliorated following prolonged immobilization with suspension. In Group B (the plate-cement group), one case suffered from plate fracture at 6 months postoperatively and underwent revision surgery; two cases developed radial nerve palsy, and the symptoms persisted at the 3-month follow-up examination. No mechanical failure of internal fixation or prosthesis, nor local tumor recurrence was observed in either group at the 3-month follow-up.

## 4 Discussion

The proximal humerus is a common site for pathological fractures of the upper extremity. Due to its abundant blood supply and cancellous bone structure, tumor cells are prone to colonization, leading to progressive bone destruction and eventual pathological fractures. Such fractures not only cause severe pain and loss of shoulder joint function but also significantly impair the patient’s quality of life. The treatment strategy is more complex than that for simple traumatic fractures, requiring a balance between symptom relief, functional recovery, tumor growth control, and survival prolongation (11). The core of the treatment strategy is to provide stable mechanical fixation to relieve pain and promote early rehabilitation, while formulating targeted individualized plans based on tumor biological behavior and the patient’s systemic condition.

Currently, conservative treatment for pathological fractures of the proximal humerus due to metastatic tumors has shown limited functional recovery, so surgery remains the preferred option. The main surgical treatments are divided into three categories: intramedullary nailing and plate fixation (focused on lesion curettage and internal fixation) and prosthetic implantation (aimed at extensive tumor resection and limb reconstruction). Janssen et al. (11) reported that the revision rate of plate fixation was approximately 10%, and that of prosthetic replacement was 11%. Wedin et al. (12) compared bone cement-augmented plate fixation and intramedullary nailing and found that bone cement-augmented plate fixation was beneficial for tumor clearance and local ablation. Rovere et al. (13) showed that both intramedullary nailing and prosthetic replacement achieved good long-term recovery, with intramedullary nailing having better short-term outcomes and prosthetic replacement being superior in tumor control. Existing tudies have confirmed the irreplaceability of bone cement-augmented plate fixation and joint arthroplasty in complex humeral metastatic fractures. Although Hoellwarth et al. discussed three surgical methods, their study was limited to renal cell carcinoma, resulting in limitations in the source of primary tumors. Casadei’s team compared plate fixation combined with bone cement and prosthetic replacement, but the number of plate fixation cases was too small, leading to potential bias in the results. The applicable scenarios of plate fixation combined with bone cement and prosthetic replacement still lack direct comparison, and the choice mainly depends on anatomical factors such as the extent of bone destruction and joint involvement, without systematically integrating multidimensional variables such as tumor type and expected survival time (14). Therefore, this study focused on the direct comparison between bone cement-augmented plate fixation and joint arthroplasty, aiming to clarify the advantages, risks, and applicable populations of the two techniques through empirical analysis (15), provide precise evidence-based support for individualized treatment, and fill the current research gap.

This retrospective comparative analysis showed that only 1 case of plate breakage required revision in our team. The bone cement-augmented plate group exhibited superior short-term functional recovery, with rapid pain relief and faster recovery of active shoulder joint range of motion and activities of daily living (16). This advantage is attributed to its minimal surgical trauma, strong immediate biomechanical stability provided by the internal fixation-bone cement complex, and maximum preservation of the native anatomical structure of the shoulder joint. In contrast, the prosthetic implantation group required extensive resection of the tumor and involved soft tissues, resulting in significant surgical trauma. Additionally, the integration and functional adaptation between the prosthesis and the host’s rotator cuff required a longer time, leading to relatively delayed short-term functional scores (16). However, the prosthetic group showed a theoretical advantage in tumor control, with a significantly lower local recurrence rate than the plate-cement group. This fundamentally stems from the surgical concept of “extensive resection,” which eliminates residual tumor cells at the source by en bloc or segmental resection of the tumor and surrounding normal tissues (17). In contrast, plate-cement fixation is essentially an intralesional procedure aimed at stabilizing the bone rather than curing the tumor, and residual tumor tissue becomes a potential source of local recurrence in the future. Differences in the complication profiles between the two groups further confirm the characteristics of the techniques. Complications in the plate-cement group, such as bone cement leakage and late screw loosening, are mainly related to material properties and surgical techniques, which can be prevented and controlled by optimizing bone cement viscosity, injection pressure, and selecting locking plate systems (15). The prosthetic group faces a higher risk of infection and nerve injury, which is associated with extensive surgical scope, prolonged operation time, impaired immune function of patients, and interference with important nerve anatomical pathways. Therefore, meticulous microsurgical techniques, strict sterile procedures, and comprehensive perioperative management are emphasized (18).

Based on these findings, we propose the following individualized clinical decision-making pathway: For patients with a short expected survival time (defined as <6 months in our team), poor systemic condition, and primary goals of palliative analgesia and rapid recovery of basic functions, plate fixation combined with bone cement augmentation is the preferred option. For patients with a long expected survival time (≥6 months), relatively slow-growing primary tumors, and extensive bone destruction, and good physical condition, tumor resection combined with prosthetic reconstruction should be preferred to achieve more durable local control and better medium-to-long-term functional outcomes (2). When the tumor has severely invaded the rotator cuff, prosthetic reconstruction is even the only option to restore partial shoulder joint function (19).

This study has several limitations (20): This study has several limitations: (1) It is a retrospective study with a small sample size, which may introduce selection bias (21); (2) The mean follow-up duration (13 months) limits our ability to evaluate long-term mechanical failure or late tumor recurrence (22); (3) The choice of surgical method was not randomized but based on surgeon preference and patient condition (23).

## 5 Conclusion

Plate fixation combined with bone cement augmentation demonstrates significantly superior short-term functional recovery, better early pain relief, and reduced perioperative trauma compared to hemiarthroplasty. However, the non-randomized nature of our surgical approach introduces selection bias, which must be considered when interpreting these results. Ultimately, surgical decision-making should be carried out depending on a comprehensive oncological evaluation.

## Data Availability

The original contributions presented in the study are included in the article/supplementary material, further inquiries can be directed to the corresponding author.
